# Whole-Body Mapping of Spatial Acuity for Pain and Touch

**DOI:** 10.1002/ana.24179

**Published:** 2014-06-06

**Authors:** Flavia Mancini, Armando Bauleo, Jonathan Cole, Fausta Lui, Carlo A Porro, Patrick Haggard, Gian Domenico Iannetti

**Affiliations:** 1 Department of Neuroscience, Physiology, and Pharmacology, University College LondonLondon, United Kingdom; 2 Institute of Cognitive Neuroscience, University College LondonLondon, United Kingdom; 3 Department of Biomedical, Metabolic, and Neural Sciences, University of Modena and Reggio EmiliaModena, Italy; 4 Department of Clinical Neurophysiology, Poole Hospital and University of BournemouthPoole, United Kingdom

## Abstract

**Objective:**

Tactile spatial acuity is routinely tested in neurology to assess the state of the dorsal column system. In contrast, spatial acuity for pain is not assessed, having never been systematically characteri**z**ed. More than a century after the initial description of tactile acuity across the body, we provide the first systematic whole-body mapping of spatial acuity for pain.

**Methods:**

We evaluated the 2-point discrimination thresholds for both nociceptive-selective and tactile stimuli across several skin regions. Thresholds were estimated using pairs of simultaneous stimuli, and also using successive stimuli.

**Results and interpretation:**

These two approaches produced convergent results. The fingertip was the area of highest spatial acuity, for both pain and touch. On the glabrous skin of the hand, the gradient of spatial acuity for pain followed that observed for touch. On the hairy skin of the upper limb, spatial acuity for pain and touch followed opposite proximal–distal gradients, consistent with the known innervation density of this body territory. Finally, by testing spatial acuity for pain in a rare participant completely lacking Aβ fibers, we demonstrate that spatial acuity for pain does not rely on a functioning system of tactile primary afferents. This study represents the first systematic characterization of spatial acuity for pain across multiple regions of the body surface. Ann Neurol 2014;75:917–924

The ability to discriminate 2 stimuli close in space, called spatial acuity is a fundamental function of exteroceptive sensory systems. Typically, spatial acuity is not homogeneous across the receptive surface, depending upon receptive field (RF) size and innervation density.^1^ In the somatosensory system, there is detailed knowledge about the topographical distribution of spatial acuity for touch throughout the whole body.^2^,^3^ This information is clinically relevant, as tactile acuity is routinely tested in neurological patients to assess the state of the dorsal column system. Reduced tactile acuity for specific body territories is the hallmark of important clinical conditions (eg, the stocking and glove distribution of impaired acuity in polyneuropathies).

In contrast, more than a century after the first description of the spatial acuity for touch across the body,^2^,^3^ it is still not known how acuity for pain is distributed throughout the body surface.

Technical difficulties in delivering sensory stimuli that are both nociceptive-selective and spatially specific^4^ underlie this knowledge gap. Radiant heat laser pulses, which excite intraepidermal Aδ- and C-fiber endings without coactivating mechanoreceptors,^5^ are commonly delivered using beam diameters of 4 to 7mm. However, it is possible to narrow the laser beam to much smaller diameters.

Here, we used this approach to provide the first systematic characterization of nociceptive spatial acuity across the body surface. We delivered 2 Nd:YAP laser pulses with a diameter of 1.3mm, and we assessed spatial acuity by measuring the 2-point discrimination (2PD) thresholds for pain across several skin regions. In 2 separate experiments conducted in healthy participants, we evaluated 2PD thresholds using both simultaneous and successive pairs of somatosensory stimuli. In each experiment, we compared 2PD for pain to 2PD for touch in the same volunteers and body sites (Fig 1). Moreover, we studied spatial acuity for pain in a rare participant completely lacking large-myelinated sensory fibers, but with intact Aδ- and C-fiber function,^6^,^7^ to test whether the measures of spatial acuity for pain were dependent on the presence of an intact tactile sensory system.

**FIGURE 1:**
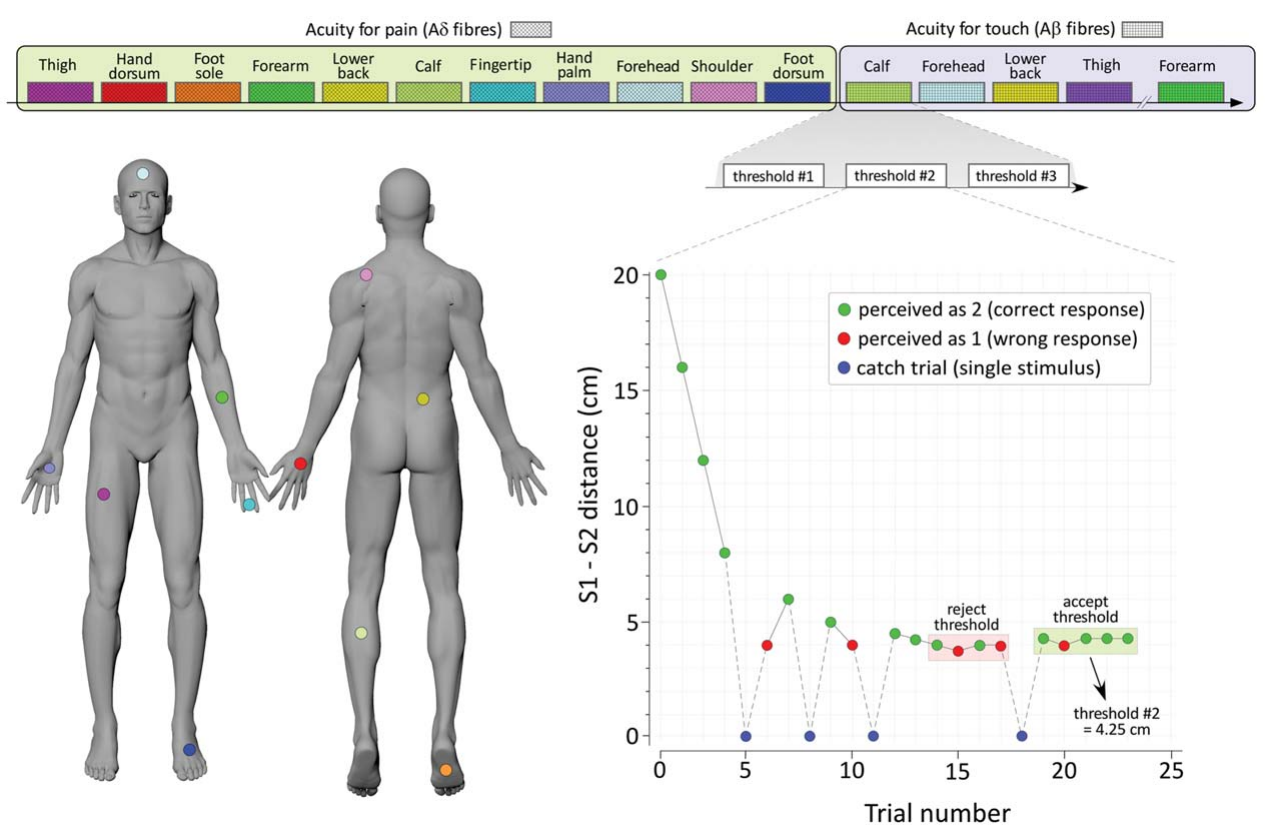
Method. Spatial acuity was assessed by measuring 2-point discrimination (2PD) thresholds for both pain and touch in 11 body territories of the same healthy volunteers. In the bottom-right panel, an example of staircase^13^ with simultaneous stimuli is depicted. For each modality, we delivered either single stimuli (25% of trials) or 2 simultaneous stimuli (75% of trials). Participants were required to discriminate whether they felt 1 or 2 stimuli, and the 2PD threshold was defined 3× for each territory, using the method of limits.

## Subjects and Methods

### Participants

Twenty-six healthy volunteers participated in the study, after having given written informed consent. Sixteen volunteers took part in Experiment 1 (9 females; mean age ± standard deviation [SD] = 23 ± 2.8 years), and 10 volunteers participated in Experiment 2 (6 females; mean age ± SD = 22.9 ± 3.3 years). The study was conducted in accordance with the principles of the Declaration of Helsinki, and was approved by the local ethics committee.

We included 1 additional participant with a rare large-fiber sensory neuropathy, consisting of a complete loss of large-myelinated Aβ fibers below the neck, but sparing thinly myelinated Aδ and unmyelinated C fibers.^6^,^7^ This 61-year-old man developed an acute sensory neuronopathy about 40 years ago, thought to follow viral diarrhea, leaving him without any sense of touch or proprioception below the neck (C3). His clinical characteristics and electrophysiological findings have been described in several single-case studies.^6^–^9^

### Nociceptive Stimuli

Noxious radiant-heat stimuli were generated by 2 identical infrared Nd:YAP lasers with a wavelength of 1.34μm (Electronic Engineering, Florence, Italy). The laser pulses were transmitted through optic fibers, and focused by lenses to a spot with a diameter of 1.3mm (approximately 1.3mm^2^; 4-millisecond duration). He-Ne lasers pointed to the area to be stimulated. The laser energy (0.35–0.46J/mm^2^) was adjusted in each subject and stimulated district to: (1) elicit a clear pinprick sensation, reflecting Aδ-fiber activation^10^; (2) achieve a mean pain intensity rating of 3 (0 = no pinprick pain, 1 = pinprick pain threshold, 10 = worst pinprick pain imaginable); and (3) match the perceived pain intensity across body territories. We allowed pain ratings to vary by ±1 score between individuals, and ±0.5 within individuals, across the explored body regions.

The skin temperature of the stimulated area was monitored during every threshold measurement with an infrared thermometer, and kept at approximately 32 ± 1°C.

In the participant without Aβ fibers, we delivered Nd:YAP laser pulses at an energy level of 0.9J/mm^2^, 0.1J/mm^2^ above detection threshold for each explored region.

### Tactile Stimuli

To assess 2PD for touch, we manually delivered somatosensory stimuli using von Frey filaments (diameter = 0.4mm, weight = 1g) mounted on an electronic vernier caliper. Stimulus duration was 1 second. In all healthy participants, these stimuli elicited a clear tactile percept, which was never described as painful and was comparable in perceived intensity.^11^

The participant with Aβ-fiber loss did not perceive any stimulus delivered with the von Frey hair (range = 0.008–300g) on the hand dorsum, palm, and fingertip, confirming that he was totally devoid of tactile sensitivity.^7^,^12^

### Procedure

In a first experiment in 16 healthy volunteers (Experiment 1), we measured 2PD using simultaneous stimuli. We randomly delivered either single stimuli (25% of trials) or 2 simultaneous stimuli (75% of trials). Participants reported whether they felt 1 or 2 stimuli. Importantly, we varied the intensity of the single nociceptive stimuli, so that some of them had a much higher intensity than the intensity of the 2 simultaneous stimuli. Therefore, the participant could not use the perceived intensity of the laser pulses as a cue to resolve the spatial task.

In a second experiment in 10 healthy volunteers (Experiment 2), we measured 2PD using successive stimuli. Each trial involved 2 stimuli, separated by an interval of 3 seconds. The first stimulus was located either more proximally or more distally than the second stimulus, with equal probability of occurrence. The task was to judge whether the second stimulus was proximal or distal relative to the first one.

In both experiments, somatosensory stimuli were delivered to 11 body regions, in separate sessions over the course of a week, at similar day times. Each session involved either tactile or nociceptive stimulation exclusively, and tested 3 or 4 randomly selected body regions in separate blocks. Throughout each session, participants lay on a bench, wearing a blindfold.

The explored body regions (see Fig 1) included: (1) the first trigeminal division on the forehead; (2) the dorsal aspect of the shoulder, about 3 to 5cm laterally to the C5 and C6 vertebral spinous processes; (3) the volar surface of forearm; (4) the hand dorsum; (5) the hand palm; (6) the volar surface of the index and middle fingertips; (7) the lower back, about 3 to 5cm laterally to the T10 and T11 vertebral spinous processes; (8) the midportion of the anterior shaft of the thigh; (9) the midcalf; (10) the foot dorsum; and (11) the inner side of the foot sole. When 2 stimuli were delivered, they were aligned along the proximal–distal axis of the body region studied, whereas the single stimulus was randomly delivered within the same skin area. The sole of the foot was not tested in Subject 1 in Experiment 1, and the forehead was not tested in Subject 2 in Experiment 2.

To measure discrimination thresholds, we used the method of limits with interleaved ascending and descending staircases (see Fig 1). In ascending staircases, the initial distance was 0.2cm. In descending staircases, the initial distance was the maximal achievable for the explored body territory. The distance between the 2 stimuli was initially adjusted in steps of 3cm, and then progressively reduced until the minimal distance at which the stimuli were correctly discriminated on 3 consecutive stimulations was reached.^13^ This distance was defined as the spatial discrimination threshold.

To avoid fatigue or sensitization of skin receptors, the stimulus locations were slightly varied from trial to trial, and the same spot was never stimulated twice within 1 minute. Threshold measurements were alternated on homologous regions of the right and left side of the body part studied, so that the same side was never stimulated in 2 consecutive blocks. In each participant, the 2PD threshold of a given body region was the average between 2 thresholds obtained on 1 side and 1 threshold obtained on the opposite side. The order of stimulated sides was balanced across participants.

Spatial discrimination in the participant with complete Aβ-fiber loss was assessed using pairs of successive stimuli, on 3 hand regions in a single session: the hand dorsum, palm, and the volar surface of the tips of the index and middle fingers. The spatial discrimination threshold was defined with the method of limits described above, using 3 staircases per body region.

## Results

**FIGURE 2:**
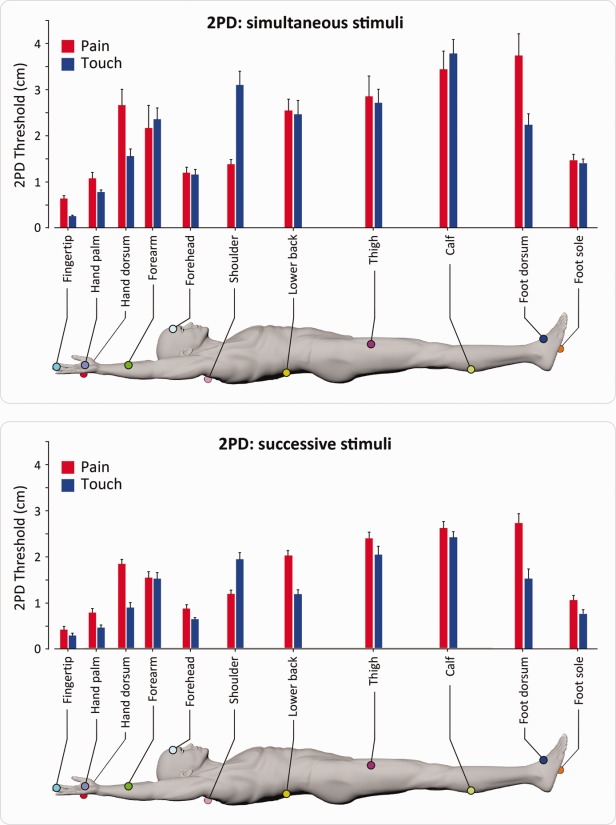
Mean 2-point discrimination (2PD) thresholds for pain and touch across the body surface. Thresholds were measured in 2 separate groups of participants, using either simultaneous stimuli (top panel, n = 16) or successive stimuli (bottom panel, n = 10). Error bars express variability (standard error) across participants. [Color figure can be viewed in the online issue, which is available at www.annalsofneurology.org.]

**FIGURE 3:**
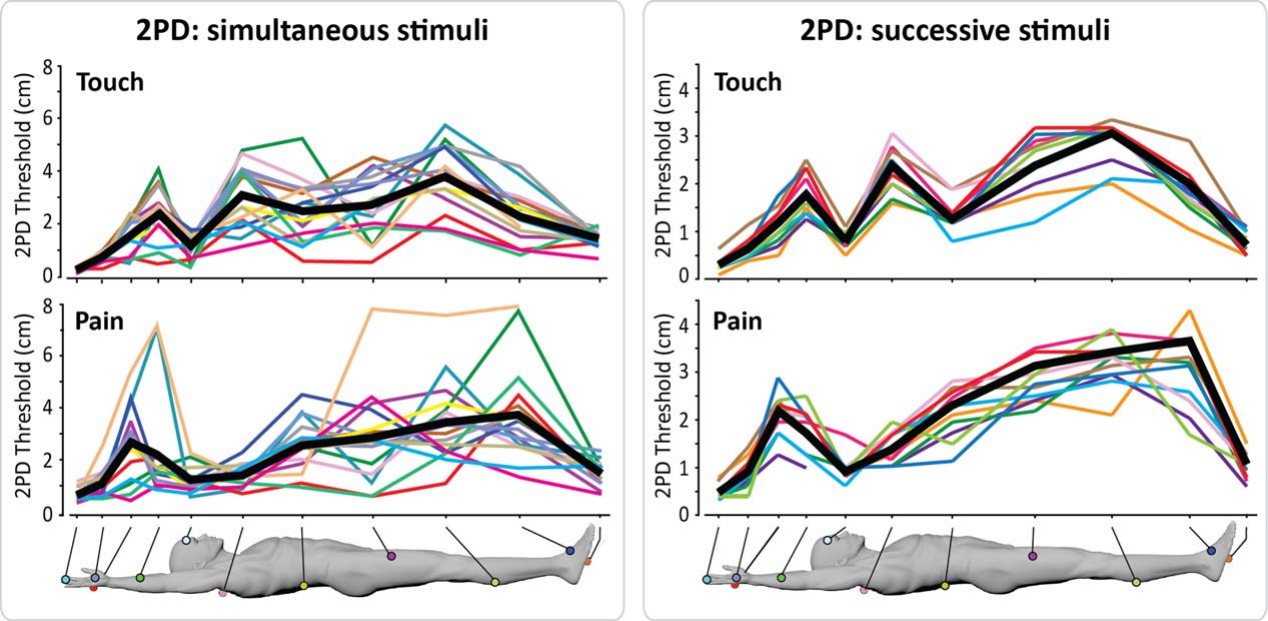
Individual 2-point discrimination (2PD) thresholds for touch (top panels) and pain (bottom panels) across the body surface, using either simultaneous (left panels, n = 16) or successive stimuli (right panels, n = 10). Thin lines depict single participants. The thick black line represents the group average. [Color figure can be viewed in the online issue, which is available at www.annalsofneurology.org.]

**FIGURE 4:**
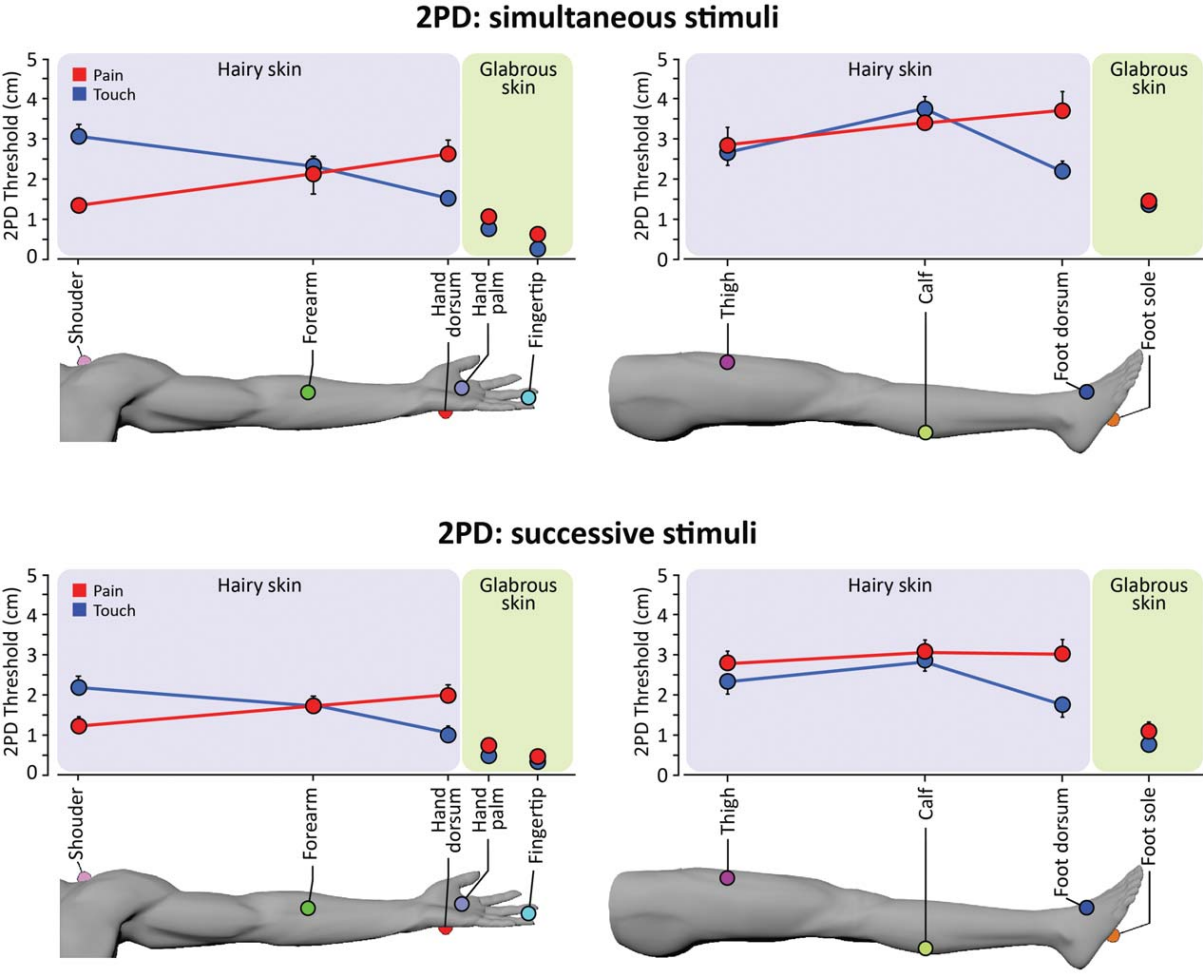
Gradients of 2-point discrimination (2PD) thresholds on the hairy and glabrous skin of the upper (left panels) and lower limbs (right panels), for pain and touch. 2PD was assessed using either simultaneous stimuli (top panels, n = 16) or successive stimuli (bottom panels, n = 10), in separate experiments. Error bars express variability (standard error) across participants. [Color figure can be viewed in the online issue, which is available at www.annalsofneurology.org.]

Figure 2 shows a group-average map of 2PD thresholds for pain and touch throughout the body surface, using both simultaneous and successive stimuli. Figure 3 displays the same thresholds for individual participants. The 2 approaches used to characterize spatial acuity (pairs of simultaneous or successive stimuli) produced convergent results. The glabrous skin of the hand and the forehead were the areas of highest spatial acuity, for both pain and touch. The gradients of spatial acuity for pain and touch were similar on the glabrous skin of the hand, whereas they followed opposite proximal–distal patterns on the hairy skin of the upper limb (Fig 4).

**FIGURE 5:**
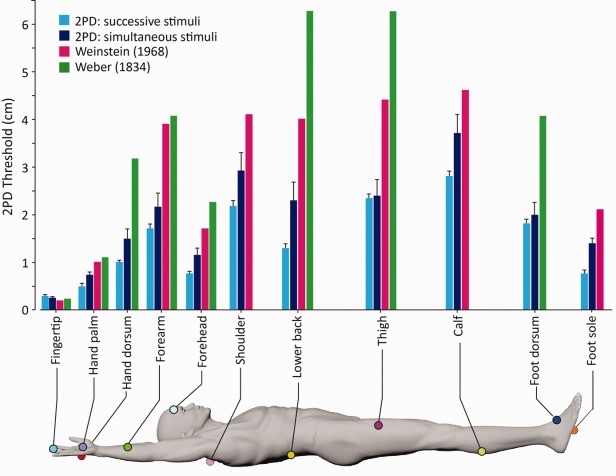
Comparison of 2-point discrimination (2PD) for touch, as measured by Weinstein,^2^ by Weber,^3^ and in the present study. Both Weinstein and Weber used simultaneous stimuli. In the present study, we used both simultaneous and successive stimuli. Error bars express variability (standard error) across participants. [Color figure can be viewed in the online issue, which is available at www.annalsofneurology.org.]

Our map of tactile acuity across the whole body follows a spatial pattern similar to that observed previously.^2^,^3^ A comparison between the current and previous results is shown in Figure 5.

### Gradients of Spatial Acuity for Pain and Touch: Hairy Skin

On the hairy skin of the upper limb, spatial acuity for pain and touch had opposite gradients. Spatial acuity for pain decreased from proximal to distal regions, whereas spatial acuity for touch increased (see Figs 2, 3, and 4). Mean individual thresholds were submitted into 2 repeated measures analyses of variance (ANOVAs) with body region (shoulder, forearm, hand dorsum) and modality (pain, touch) as experimental factors, separately for 2PDs measured using simultaneous and successive stimuli. There was a highly significant body region by modality interaction for both simultaneous (*F*_2,30_ = 23.8, *p* < 0.0001) and successive 2PD (*F*_2,18_ = 93.8, *p* < 0.0001), but no main effect of either factor (simultaneous 2PD: both factors, *F* < 1; successive 2PD: body region, *F*_2,18_ = 3.0, *p* = 0.075; modality, *F* < 1).

To explore these interactions, we performed 2 follow-up 1-way ANOVAs, 1 for each modality, with body region (shoulder, forearm, hand dorsum) as experimental factor. We also performed a contrast for proximal–distal differences to test the a priori hypothesis of a difference on spatial acuity between the most proximal and distal regions of each limb. All analyses were performed separately for simultaneous and successive 2PD. The 1-way ANOVAs revealed a significant main effect of body region, for both pain (simultaneous 2PD, *F*_2,30_ = 4.62, *p* = 0.018; successive 2PD, *F*_2,18_ = 20.6, *p* < 0.0001) and touch (simultaneous 2PD, *F*_2,30_ = 19.35, *p* < 0.0001; successive 2PD, *F*_2,18_ = 48.62, *p* < 0.0001). The proximal–distal contrasts (shoulder vs hand dorsum) were also significant for both simultaneous 2PD (pain, *t*_15_ = −3.91, *p* = 0.001; touch, *t*_15_ = 6.37, *p* < 0.0001) and successive 2PD (pain, *t*_9_ = −5.57, *p* < 0.0001; touch, *t*_9_ = 11.03, *p* < 0.0001). Critically, the linear functions for pain and touch had opposite slopes (see Fig 4), indicating that spatial acuity for touch linearly increases from proximal to distal regions, whereas that for pain linearly decreases from proximal to distal regions.

On the lower limb, an ANOVA with body region (thigh, calf, foot dorsum) and modality (pain, touch) as experimental factors revealed a significant effect of body region (simultaneous 2PD, *F*_2,30_ = 3.6, *p* = 0.040; successive 2PD, *F*_2,18_ = 5.62, *p* = 0.013). The effect of modality was significant only for successive 2PD (*F*_1,9_ = 31.29, *p* < 0.0001), but not for simultaneous 2PD (*F*_1,15_ = 1.2, *p* = 0.289). The interaction between body region and modality was again highly significant for both tasks (simultaneous 2PD, *F*_2,30_ = 8.1, *p* = 0.002; successive 2PD, *F*_2,18_ = 10.09, *p* = 0.001). Thus, we performed the same follow-up analyses as for the upper limb. The main effect of body region was not significant for pain (simultaneous 2PD, *F*_2,30_ = 1.7, *p* = 0.195; successive 2PD, *F*_2,18_ = 1.62, *p* = 0.226), but was significant for touch (simultaneous 2PD, *F*_2,30_ = 14.5, *p* < 0.0001; successive 2PD, *F*_2,18_ = 19.53, *p* < 0.0001). This suggests that on the hairy skin of the lower limb there is a gradient of spatial acuity for touch, but not for pain (see Fig 4). The contrasts for proximal–distal differences (thigh vs foot dorsum) were not significant for pain (simultaneous 2PD, *t*_15_ = −1.56, *p* = 0.14; successive 2PD, *t*_9_ = −1.76, *p* = 0.112). There was a significant proximal–distal difference for the discrimination of successive tactile stimuli (*t*_9_ = 3.02, *p* = 0.015), but not for the discrimination of simultaneous tactile stimuli (*t*_15_ = 1.58, *p* = 0.13).

### Gradients of Spatial Acuity for Pain and Touch: Glabrous Skin

To investigate the gradient of spatial acuity on the glabrous skin, we performed 2 ANOVAs with hand region (palm, fingertip) and modality (pain, touch) as factors, separately for the 2 tasks. Although 2PD thresholds were overall lower for touch than for pain (modality: simultaneous 2PD, *F*_1,15_ = 21.5, *p* < 0.0001; successive 2PD, *F*_1,9_ = 16.60, *p* = 0.003), there was a significant gradient of spatial acuity on the glabrous skin of the hand (hand region: simultaneous 2PD, *F*_1,15_ = 42.2, *p* < 0.0001; successive 2PD, *F*_1,9_ = 39.05, *p* < 0.0001), with lower 2PDs on the fingertip than on the palm (see Figs 2, 3, and 4). There was a significant hand region by modality interaction only for successive 2PD (*F*_1,9_ = 17.21, *p* = 0.002), but not for simultaneous 2PD (*F* < 1). The difference between discrimination thresholds of successive points on the palm and fingertip was significant for both pain (*t*_9_ = 6.10, *p* < 0.0001) and touch (*t*_9_ = 5.06, *p* = 0.001). We directly tested whether the spatial gradients of acuity on the glabrous skin of the hand were comparable across the 2 modalities, by calculating the ratio between 2PD thresholds on the finger and on the hand palm. This ratio was greater for touch than for pain (simultaneous 2PD, *t*_15_ = 3.83, *p* = 0.002; successive stimuli, *t*_9_ = 3.40, *p* = 0.008).

### Comparing Spatial Acuity in Glabrous and Hairy Skin

We performed 2 separate ANOVAs, 1 for the hand and 1 for the foot, to investigate differences between hairy and glabrous skin. The experimental factors were skin type (dorsum, palm [for hand]; or: dorsum, sole [for foot]) and modality (pain, touch).

On the hand, 2PD thresholds were overall significantly lower on the palm than on the dorsum (skin type: simultaneous 2PD, *F*_1,15_ = 52.55, *p* < 0.0001; successive 2PD, *F*_1,9_ = 69.01, *p* < 0.0001). 2PD thresholds were also lower for touch than for pain (modality: simultaneous 2PD, *F*_1,15_ = 11.62, *p* = 0.004; successive 2PD, *F*_1,9_ = 84.02, *p* < 0.0001). Finally, there was a significant skin type by modality interaction for both tasks (simultaneous 2PD, *F*_1,15_ = 7.19, *p* = 0.017; successive 2PD, *F*_1,9_ = 37.32, *p* < 0.0001). The ratios between 2PD thresholds on the hand palm and dorsum were higher for touch than for pain only in the successive 2PD task (*t*_9_ = 2.40, *p* = 0.041), whereas they were comparable in the simultaneous 2PD (*t*_15_ < 1, *p* = 0.562; see Fig 5).

We observed a similar pattern of results on the foot. The ANOVA revealed significant main effects of skin type (simultaneous 2PD, *F*_1,14_ = 46.3, *p* < 0.0001; successive 2PD, *F*_1,9_ = 146.98, *p* < 0.0001) and modality (simultaneous 2PD, *F*_1,14_ = 8.3, *p* = 0.012; successive 2PD, *F*_1,9_ = 21.61, *p* = 0.001). The interaction between these factors was also significant (simultaneous 2PD, *F*_1,14_ = 4.5, *p* = 0.052; successive 2PD, *F*_1,9_ = 22.34, *p* = 0.001). Again, the ratios between 2PD thresholds on the foot sole and dorsum were higher for touch than for pain in the successive 2PD (*t*_9_ = 3.74, *p* = 0.005), but not in the simultaneous 2PD task (*t*_14_ = 1.62, *p* = 0.127).

### Spatial Acuity for Pain and Touch on the Forehead

On the forehead, 2PD thresholds for pain and touch were similar for both simultaneous 2PD (mean difference [pain − touch] ± standard error [SE], 0.04 ± 0.17cm; paired *t* test: *t*_15_ = 0.226, *p* = 0.824) and successive 2PD (mean difference [pain − touch] ± SE, 0.25 ± 0.13cm; paired t-test: *t*_8_ = 1.89, *p* = 0.096), as shown in Figure 2.

### Participant with Complete Loss of Aβ Fibers

The spatial discrimination thresholds for suprathreshold pinprick stimuli were overall similar to those observed in healthy individuals (see Figs 2 and 3). On the glabrous skin, spatial discrimination thresholds for successive stimuli were identical (ie, 0.8 ± 0.3cm) for both palm and fingertip. Similarly to neurologically unimpaired participants, thresholds for successive 2PD were higher on the hairy skin (mean ± SD, 1.8 ± 1.2cm) than on the glabrous skin of the hand.

## Discussion

Variations in tactile spatial acuity across the body have long been known.^2^,^3^ Our study represents the first systematic characterization of spatial acuity for pain across multiple regions of the body surface. The possibility of precisely mapping acuity for pain is clinically important for a number of reasons. These include assessing the function of small-fiber impairment in neuropathies, as part of quantitative sensory testing,^14^ as well as studying the mechanisms of neural plasticity in the nociceptive system,^15^,^16^ of which spatial acuity is the most obvious behavioral marker.^17^–^19^

We measured spatial discrimination thresholds using either simultaneous or successive stimuli. It is worth noting that thresholds obtained using 2 successive stimuli are usually lower than those obtained using 2 simultaneous stimuli,^20^ because of the extra spatial information contained in the differential activation of partially overlapping RFs when a single stimulus at a time is applied.^21^ We did observe that 2PDs obtained using successive stimuli were overall lower than 2PD measured using simultaneous stimuli (see Figs 2 and 3). However, the 2 methods yielded largely convergent results in relation to the topographical differences in spatial acuity across body territories (see Figs 2, 3, and 4).

The glabrous skin of the hand and the forehead were the areas of highest spatial acuity, regardless of the method used (pairs of successive or simultaneous stimuli), for both touch and pain. Remarkably, in these regions, spatial acuity for pain approaches the exquisite spatial precision repeatedly observed for touch. In the remaining body regions, 2PD for pain was often poorer than 2PD for touch.

### Gradients of Spatial Acuity on the Hairy Skin of the Upper Limb Follow Innervation Density

Previous investigations of spatial acuity for nociceptive-selective stimulation compared acuity for pain and touch in single body territories, without investigating spatial gradients for pain across body sites.^20^,^22^,^23^ In the present study, we replicated the observation of a topographical distribution of spatial acuity for touch on the hairy skin of the upper limb, with better acuity distally than proximally (see Fig 5).^2^,^24^ By delivering nociceptive selective stimuli to the same sites, we also detected, for the first time, a clear gradient of spatial acuity for pain. This gradient for pain had an opposite direction compared to touch, with better acuity proximally than distally on the hairy skin of the upper limb (see Fig 4).

The opposite gradients of spatial acuity on the upper limb reflect the known innervation density and RF size of mechanoreceptors and nociceptors. The density of skin nociceptors in the back and the neck is higher than in the hand dorsum,^25^ and more generally, intraepidermal innervation is reported to be denser proximally than distally.^26^–^28^ In contrast, mechanoreceptor density reportedly shows the opposite distribution.^1^,^29^,^30^

The density of peripheral receptors innervating a given portion of the receptive surface influences the size of the area of the primary sensory cortex devoted to process the sensory input.^1^ The somatosensory homunculus mapped by Penfield in the primary somatosensory cortex (SI) has an iconic status in neuroscience; it shows a characteristic increase in cortical representation for distal compared to proximal tactile inputs, consistent with the gradient of tactile acuity described both here and previously.^2^,^3^ In contrast, a systematic evaluation of cortical magnification for nociceptive input across the body surface is still missing.

### Both Pain and Touch Have Maximal Spatial Acuity on Glabrous Skin

For both pain and touch, spatial acuity was higher on the fingertip than on the hand palm. These findings confirm and extend previous evidence of a fovea for pain at the fingertips.^31^

Using successive nociceptive stimuli, we had already observed that spatial acuity was higher on the fingertips than on the hand dorsum, for both pain and touch. Spatial discrimination thresholds obtained using successive stimuli on the fingertips were 0.5cm (SE ± 0.6) and 0.2cm (SE ± 0.4) for pain and touch, respectively.^31^ Here, using both simultaneous and successive stimuli, we report similar thresholds (see Figs 2, 3, and 4).

Crucially, in our previous study^31^ we quantified the intraepidermal nerve fiber density (IENFD), by performing skin biopsies. Remarkably, IENFD was lower on the fingertips than on the hand dorsum, so the spatial gradient for pain acuity must presumably be explained by some other, central factors.^31^ In the current study we confirm the finding of an area of highest spatial acuity for both pain and touch on the fingertips, and demonstrate that spatial acuity on the palm is worse than on the fingertips. Furthermore, we show that spatial acuity for pain and touch is overall higher on glabrous skin than on hairy skin, for both the hand and the foot (see Figs 2, 3, and 4).

Probably related to this finding is the recent discovery that the human SI contains fine-grained maps reflecting nociceptive-selective input to individual digits.^32^ These nociceptive maps are highly aligned to maps of tactile input to the same digits, in each individual subject. Because tactile acuity is related to cortical magnification in SI,^33^ we suggest that the remarkable cortical magnification of nociceptive signals in SI is the likely neuronal correlate of the high spatial resolution for pain on the glabrous skin of the hand. However, it remains unclear what transformation, at the spinal or cortical level, could subserve the cortical magnification of nociceptive inputs from the glabrous skin. Furthermore, it should be noted that other somatotopically organized, fine-grained nociceptive maps might exist in other cortical areas (eg, the operculoinsular cortex^34^,^35^), and potentially be related to spatial acuity for pain. However, these maps have never been explicitly looked for. The finding of a preserved spatial acuity in the participant with complete Aβ-fiber loss demonstrates that spatial acuity for pain does not rely on a functioning system of tactile primary afferents.
